# Primary glomus tumor of the thymus in a 66-year-old patient

**DOI:** 10.1186/s13019-024-02806-8

**Published:** 2024-06-10

**Authors:** Yibing Zang, Ruixing Zhao, Chengquan Ma, Dejun Gao

**Affiliations:** 1 Department of Thoracic Surgery, the Second People’s Hospital of Liaocheng, 306 Health Street, Linqing City, Shan Dong, 252600 China; 2 Department of Urology, Tianjin Medical University General Hospital, Tianjin, 300052 China

**Keywords:** Glomus tumor, Thymus, Thoracoscopic thymectomy, Smooth muscle actin

## Abstract

We report a unique case of a 66-year-old man who was incidentally identified to have a mass in the thymus region by computerized tomography scan. CT revealed a well-defined 1.6 × 1 × 0.9 cm thymus mass with moderate uniform enhancement. Thoracoscopic thymectomy was performed, and the pathological diagnosis was primary glomus tumor of the thymus. There were no atypia or malignant histological features, and no primary tumors in other sites. To our knowledge, this is the first case of primary thymic glomus tumor reported in the literature.

## Introduction

Glomus tumor (GT) occurs mostly in the distal part of the limb, such as the nail bed and the finger (toe) side [1], and is rare in the internal organs and deep soft tissues. The vast majority of GT were benign with only 1% being malignant [2]. Previous studies reported mediastinal glomus tumor, but did not locate the thymus [3]. GT located in the thymus is rare and has not been reported in the literature.

## Case report

**Fig. 1:**
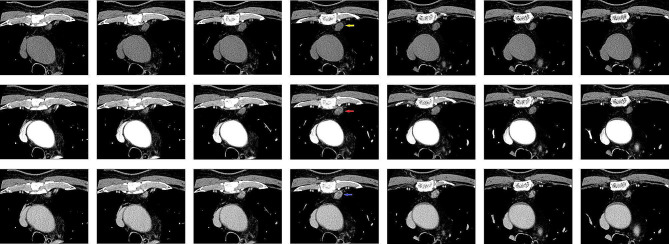
1.6 × 1 × 0.9 cm mass (Red /yellow/blue arrow) in the thymus region with CT scan. The plain CT (First line pictures) value was 25 HU, and the enhanced CT values were 34 HU (Arterial phase, Second line pictures) and 76 HU (Venous phase, Third line pictures)

A 66-year-old man was found to have a 1.3 cm mass in the thymus by CT scan during his physical examination at an external hospital one year ago, and it was recommended that he undergo CT examination and monitoring every year. He came to our hospital for further mass examination 1 month ago. He had a history of hypertension for 10 years and coronary heart disease for 5 years. He was regularly controlled with drugs and was satisfied. His lab results were all within normal limits. Chest CT showed a 1.6 × 1 × 0.9 cm mass in the anterior mediastinum with clear boundaries. The plain CT value was 25 HU, and the enhanced CT values were 34 HU (Arterial phase) and 76 HU (Venous phase), which was considered to be malignant thymoma (Fig. 1). CT of the abdomen and pelvis and bone scans were normal, with no clear evidence of metastasis.

The patient underwent thoracoscopic thymectomy. During the operation, there was no adhesion in the pleura, no abnormality in the mediastinal pleura, visceral parietal pleura and lung. The tumor was located in the anterior superior mediastinal thymus region without adhesion to the lung lobe. The thymus tissue was removed along the left mediastinal pleura along with the fat in the anterior pericardial region. The operation was successful. A firm and gray 1.6 × 1 × 1 cm tumor was found in the resected thymus tissue.

**Fig. 2:**
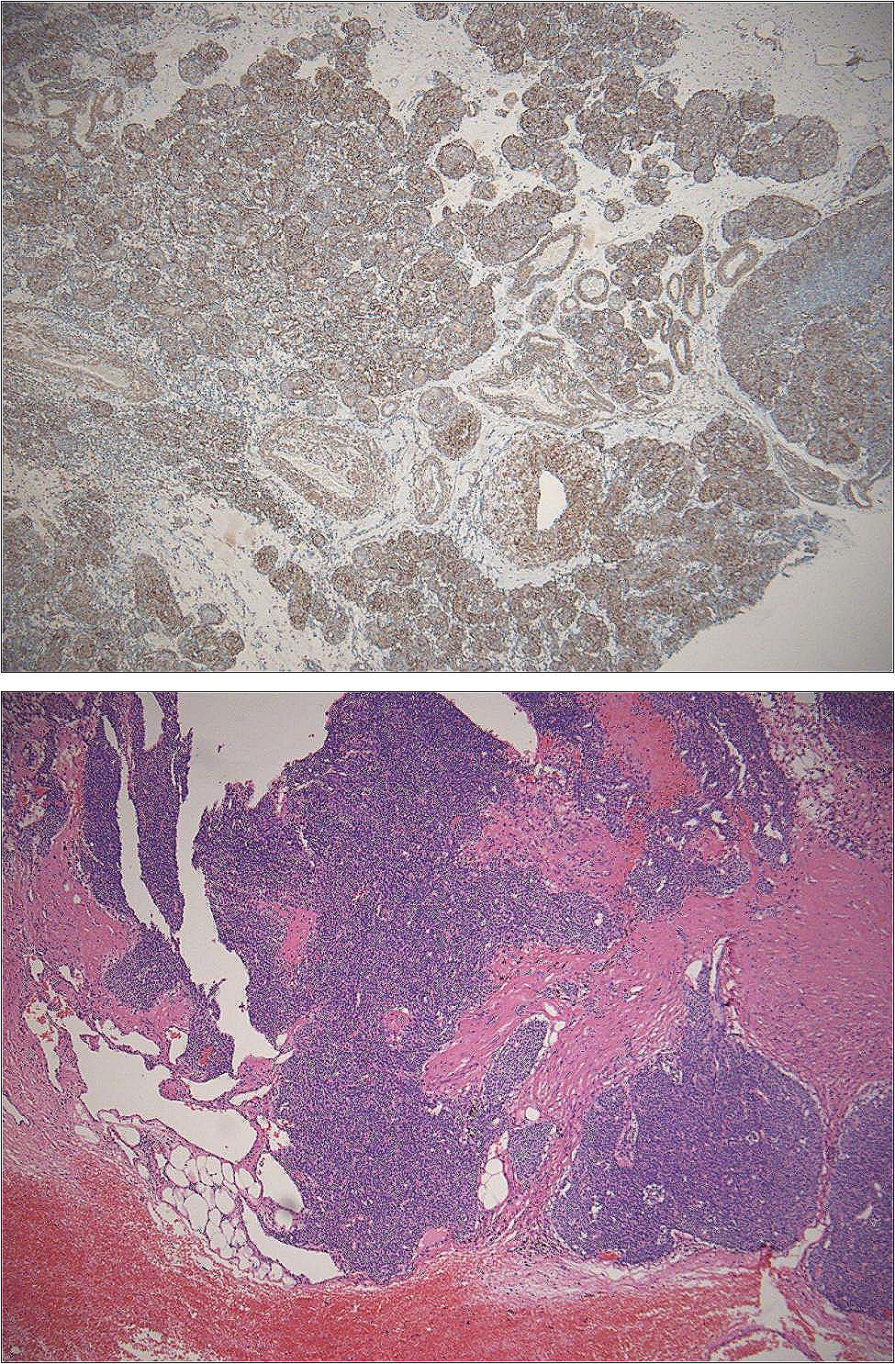
Pathological view confirmed the presence of primary glomus tumor of the thymus. The histopathological features of GT were obvious proliferation of smooth muscle cells under the microscope, and the vascular lumens were more common, and the tumor nests were surrounded by spherical cells around the blood vessels. (picture below --- H&E stain, original magnification ×40; picture above --- showing glomus cells positive for smooth muscle actin, original magnification ×40)

By histopathological examination (Fig. 2), the mass was confirmed as a primary glomus tumor of the thymus (PGTT). Immunohistochemistry were as follows: CK19(-), P63(-),CD20(-),TdT(-),CD3(-),PR(-),SMA(+),SATR2(-),INSM1(-),Syn(-),CD56(-),Ki-67(2%+).

A month after the surgery, all the lab results are all within normal limits, no post-operative complications, tumor recurrence and metastasis.

## Discussion

GT involving deep internal organs are rare, and their histogenesis and origin are largely uncertain due to the relative lack or almost absence of glomus at these sites.

GT that occurs in the extremities may have classic pain symptoms, while most that occur in internal organs have no classic symptoms. Therefore, the diagnosis of GT in the internal organs is often difficult. The most common presentation is a well-defined mass on CT, with enhanced features after enhanced scan. However, when it occurs in the thymus, it is difficult to distinguish from a thymoma. Therefore, pathological examination is essential for correct diagnosis. Morphologically, glomus tumors (GTs) are well defined and consist of different proportions of glomus cells, smooth muscles, and blood vessels. Based on the relative prominence of these three components, GTs are divided into solid GT, GT or glomus fibroids, of which solid tumors are the most common [4]. Genetically, Mosquera et al. identified changes in the Notch signaling pathway, which regulates vascular smooth muscle development, in more than half of the 33 GT they screened [5].

The main differential diagnosis of PGTT includes thymoma and thymic carcinoma. Patients with thymus cancer usually have no specific clinical manifestations and rarely have paraneoplastic syndrome. However, thymic carcinoma is a highly aggressive malignancy, with local invasion and distant metastases frequently seen. Thymomas are currently considered to have malignant potential and also require surgical removal. Immunohistochemical staining often helps to distinguish them. The immunohistochemical staining of the patient in this case was positive only for SMA, and the rest were negative, which was consistent with the immunohistochemical characteristics of glomus tumor, and thymoma and thymic malignancy were excluded.

Most GT were isolated, benign, < 1 cm in diameter, with clear boundaries, and malignant only accounted for about 1% ^2^. The histopathological features of GT were obvious proliferation of smooth muscle cells under the microscope, and the vascular lumens were more common, and the tumor nests were surrounded by spherical cells around the blood vessels. The diagnostic criteria for malignant GT included tumor > 2 cm in diameter, intermediate-high grade atypia, and mitotic images > 5/50 high power field (HPF). It has been suggested that if GTs do not meet the criteria for malignant tumors, but have at least one atypical feature in addition to pleomorphism (e.g., diameter greater than 2 cm, deep location, and no heterotypic or mitotic activity), they should be considered malignant potential [6]. In our case, no criteria for malignancy described above were found, but a diagnosis of GTs with uncertain malignant potential was established based on internal organ origin, and close long-term follow-up was recommended.

## Data Availability

No datasets were generated or analysed during the current study.
